# Patterns of ranibizumab and aflibercept treatment of central retinal vein occlusion in routine clinical practice in the USA

**DOI:** 10.1038/eye.2014.308

**Published:** 2015-01-09

**Authors:** A J Lotery, S Regnier

**Affiliations:** 1 Clinical and Experimental Sciences, Faculty of Medicine, University of Southampton, Southampton, UK; 2 Novartis Pharma AG, Basel, Switzerland

## Abstract

**Background:**

The intravitreal anti-vascular endothelial growth factor treatments ranibizumab and aflibercept have proven efficacy in clinical trials, but their real world usage in central retinal vein occlusion (CRVO) has not been assessed. We therefore evaluated the treatment patterns of both drugs in a US claims database.

**Methods:**

The IMS Integrated Data Warehouse was used to identify the patients with CRVO in the USA with claims for ranibizumab or aflibercept between 24 September 2012 and 31 March 2014 with at least 12 months follow-up. Patients were required to have had no anti-VEGF treatment code for 6 months before index (‘treatment-naive'). Mean numbers of injections and non-injection visits to a treating physician were compared with patients receiving these treatments.

**Results:**

Patient characteristics were similar for patients receiving ranibizumab (*n*=206) or aflibercept (*n*=79) at index. The mean (±SD) numbers of injections received by patients treated with ranibizumab or aflibercept were 4.4±2.8 and 4.7±2.9 (*P*=0.38), respectively; the total number of patient visits to their treating physician was 7.3±3.7 and 7.0±2.9 (*P*=0.52), respectively. For patients receiving one or more injections (*n*=238), the mean interval between injections was 55.1 days (ranibizumab) and 54.2 days (aflibercept; *P*=0.44).

**Conclusions:**

Our results suggest that, in routine clinical practice, patients receive a comparable number of injections in the first year of treatment with ranibizumab or aflibercept. This may have implications for commissioning and service development of CRVO care pathways.

## Introduction

Macular edema secondary to retinal vein occlusion (RVO) can cause severe visual impairment owing to obstruction of the retinal vasculature, and is the second most common retinal vascular disease.^1, 2^ Occlusion of the retinal veins causes an increase in retinal capillary pressure resulting in upregulation of vascular endothelial growth factor (VEGF) expression and a consequent increase in vascular permeability and new vessel proliferation within the iris and anterior chamber. As a result, blood and plasma are discharged into the retina, often causing complications including macular edema and varying degrees of ischemia, potentially leading to severe vision loss. Although occlusion of the central retinal vein (central RVO (CRVO)) occurs less frequently than in branch veins, it is associated with severe visual outcomes.

Anti-VEGF therapy is now the standard of care for CRVO, replacing the previous observation-only approach.^3, 4, 5^ Ranibizumab (Lucentis; Genentech Inc., San Francisco, CA, USA and Novartis Pharma AG, Basel, Switzerland) is a humanized, affinity-matured VEGF antibody fragment that binds to and neutralizes all isoforms of VEGF. Ranibizumab is recommended to be given monthly based on the evidence from clinical trials.^6^ The efficacy of ranibizumab for the management of CRVO has been reported in multiple studies including the Randomized Study Comparing Ranibizumab to Sham in Patients with Macular Edema Secondary to CRVO (ROCC)^7^ and the Ranibizumab for the Treatment of Macular Edema After CRVO Study (CRUISE);^8, 9^ intravitreal injections of ranibizumab provided rapid improvement in 6-month visual acuity and macular edema following CRVO, with low rates of adverse events.^7, 8^ These improvements were largely maintained with a subsequent 6 months of dosing as required (*pro re nata* (PRN)).^9^ Ranibizumab was approved for treatment of macular edema secondary to CRVO by the US Food and Drug Administration (FDA) in June 2010.^10^

Aflibercept is a fully human, recombinant fusion protein that targets VEGF-A, VEGF-B, and placental growth factor. Aflibercept binds all isoforms of VEGF-A with high affinity—a markedly higher affinity than that of ranibizumab. Like ranibizumab, aflibercept is recommended in the USA to be given as monthly intravitreal injections.^11^ Patients should subsequently be monitored regularly, and treatment should be resumed if visual outcomes deteriorate. Two recent clinical trials (VEGF Trap-Eye: Investigation of Efficacy and Safety in CRVO (GALILEO)^12, 13^ and VEGF Trap-Eye for macular edema secondary to CRVO (COPERNICUS)^14, 15^) have shown that monthly intravitreal aflibercept treatment was well tolerated and improved visual acuity after 6 months significantly more than sham injections; these improvements were maintained with subsequent monthly monitoring and PRN dosing.^12^ Aflibercept was approved for the treatment of macular edema secondary to CRVO in September 2012.^16^

Despite promising results from clinical trials as described above, real world usage of aflibercept and ranibizumab in CRVO has not yet been studied. This study therefore aimed to assess the treatment patterns of ranibizumab and aflibercept for the management of macular edema secondary to CRVO in routine clinical practice in the USA using a large, patient-level, physician-entered claims database.

## Materials and methods

This retrospective study was based on the analysis of US physician-level claims data from the Integrated Data Warehouse (IDW; managed by IMS Health, Plymouth Meeting, PA, USA), a claims database that encompasses ∼1 billion professional fee claims per year, representing ∼80% of practicing eye care specialists (including over 13 000 ophthalmologists) and covering all 50 states. Approximately 95% of claims submitted for payment from these sources are available for analysis within 3 weeks.

The study included adult patients with a first medical claim registered in the IDW with a procedure code for intravitreal injection of ranibizumab or aflibercept between 24 September 2012 and 31 March 2014, and with a concomitant diagnosis of CRVO (recorded as a code from the International Classification of Disease 9th Revision Clinical Modification; ICD-9-CM 362.35); this first claim was defined as the patient's index date. Patients were required to have at least 12 months of follow-up data (post index date) within this study period and a minimum of 6 months of available data in the IDW before the index date. The physician administering the index medication was required to have consistently submitted medical claims to the IDW during the 6 months before the index date and during the follow-up period (‘physician stability' criteria). Patients were excluded from the analysis if: their records indicated that they had received an anti-VEGF injection during 6 months before the index date (ensuring ‘naivety'); if they received more than one anti-VEGF drug within 12 months after the index date (to avoid the potential confound of a patient being included in both groups). The last assumption was relaxed in the sensitivity analysis to assess the number of any anti-VEGF injections received by patients starting on ranibizumab and aflibercept.

The primary analysis assessed the number of injections received, non-injection visits made and total visits (ie, the sum of injection and non-injection visits) made by treatment-naive patients (defined as having received no anti-VEGF treatment claim in the 6 months before the index date) who were treated continuously (ie, received no other anti-VEGF therapy) with their index therapy for at least 12 months (365 days). Mean dosing intervals (number of days between the injections) were determined for the first year of therapy for patients starting on either treatment and receiving at least two injections.

Differences between the treatment patterns of ranibizumab and aflibercept were assessed, and reported *P*-values were adjusted for baseline characteristics. Negative binomial regression was used to compare the effect of patient characteristics on injection and visit estimates for those treated continuously with ranibizumab and aflibercept for at least 12 months. A generalized estimating equation (GEE) model applied at the patient level was used to compare the effect of patient characteristics on dosing interval estimates for patients having received two or more injections. The nesting assumption is reviewed in the discussion section. Finally, an autocorrelation of order 1 was used for within-cluster correlation.

Several sensitivity analyses were performed: we assessed the mean number of injections, non-injection visits, and total visits including anti-VEGFs other than that given at index (‘any anti-VEGF'); and we assessed the first 6 months of data to see if there were between-group differences. We also assessed the baseline characteristics of the patients receiving only one injection compared with those receiving multiple injections, to assess whether this patient subset could confound the analyses. For continuous variables, between-group statistical differences were assessed using unpaired Student's *t*-tests, with *P*<0.05 used to define a significant difference. Categorical variables were assessed using Fisher's exact test.

## Results

**Figure 1:**
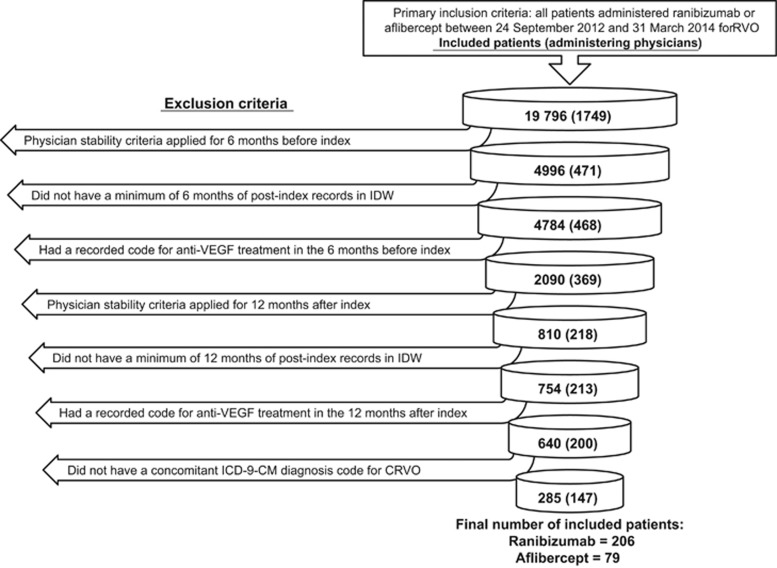
Development and attrition of patient cohorts. CRVO, central retinal vein occlusion. ICD-9-CM, International Classification of Disease 9th Revision Clinical Modification. IDW, integrated data warehouse. RVO, retinal vein occlusion. VEGF, vascular endothelial growth factor.

In total, 285 patients were treated continuously with their index drug over 12 months (ranibizumab, *n*=206; aflibercept, *n*=79; Figure 1). The two treatment groups were comparable in terms of demographics or type of health plan, and almost all patients received treatment from an ophthalmologist (including retinal specialists) (Table 1). The majority of patients in both the groups (ranibizumab, 57% aflibercept, 53%) were female, and their median (interquartile range) ages were 74.0 (67.0–81.0) years and 76.0 (70.0–81.0) years, respectively. Cancer, cardiovascular disease, chronic pulmonary disease, and diabetes mellitus were the only comorbidities listed in the Charlson–Deyo comorbidity index (CCI)^17^ that occurred in >5% of patients in either group.

**Figure 2:**
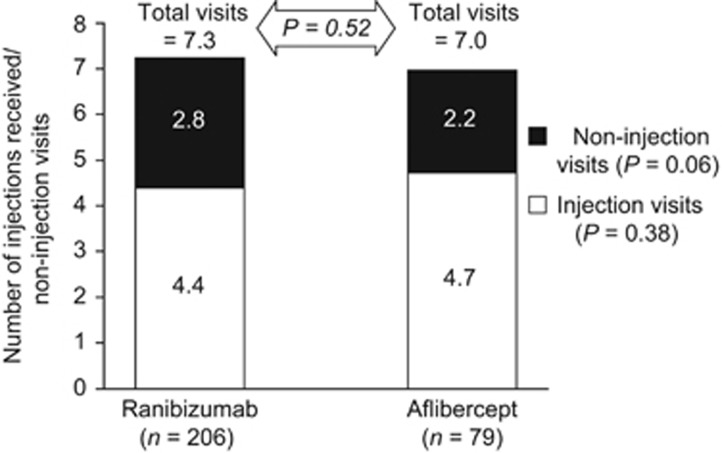
Annual mean number of injections and annual mean number of non-injection visits in the first year of therapy in patients receiving treatment with ranibizumab or aflibercept for the management of central retinal vein occlusion.

For patients treated continuously with ranibizumab or aflibercept, the mean±SD number of injection visits during the first 12 months of treatment (based on a negative binomial model adjusting for characteristics; Supplementary Table 1) was 4.4±2.8 and 4.7±2.9 (*P*=0.38), respectively, and that of non-injection visits was 2.8±2.6 and 2.2±2.1 (*P*=0.06), respectively (Figure 2). The total number of visits to the treating physician in the 12 months after the index date was 7.3±3.7 and 7.0±2.9 (*P*=0.52) for ranibizumab and aflibercept, respectively. Patients received an injection on the majority of their visits to their prescribing physician (ranibizumab, 63%±26% aflibercept, 67%±27%). For patients receiving one injection or more (*n*=238), the mean interval between the injections was 55.1 days for patients treated continuously with ranibizumab and 54.2 days for patients treated continuously with aflibercept (*P*=0.44). The details of the GEE model used to adjust for the effects of patient characteristics on mean interval length are shown in Supplementary Table 2.

**Figure 3:**
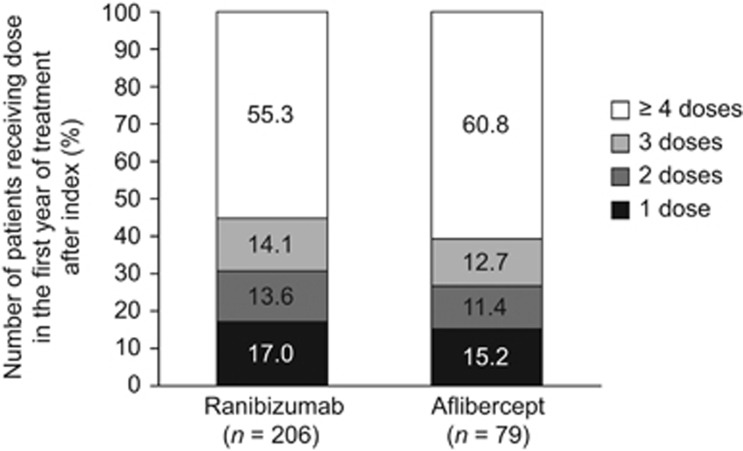
Distribution of injections received by patients starting therapy with ranibizumab or aflibercept for the management of central retinal vein occlusion during the first year of therapy.

Over half of the patients in each group had four or more injections of their index drug within the first year of treatment (ranibizumab, 55.3% aflibercept, 60.8% Figure 3). Over 40% of all patients received four doses in the first 6 months of therapy with their index treatment (ranibizumab, 40.3% aflibercept, 43.0%). Approximately 20% of all patients received five or more doses in the first 6 months of therapy with their index treatment (ranibizumab, 22.3% aflibercept, 19.0%).

When the inclusion criteria were extended to include any additional anti-VEGF treatment claims during follow-up (ranibizumab, *n*=261; aflibercept, *n*=93), the numbers of all anti-VEGF injections received in the first 12 months of follow-up were 4.7±2.7 and 4.8±2.9 (*P*=0.59) for patients starting on ranibizumab and aflibercept, respectively (when adjusting for baseline characteristics). The according number of non-injection visits were 3.0±2.6 and 2.3±2.2 (*P*<0.05) and the total number of visits were 7.6±3.6 and 7.1±2.9 (*P*=0.25), respectively.

**Figure 4:**
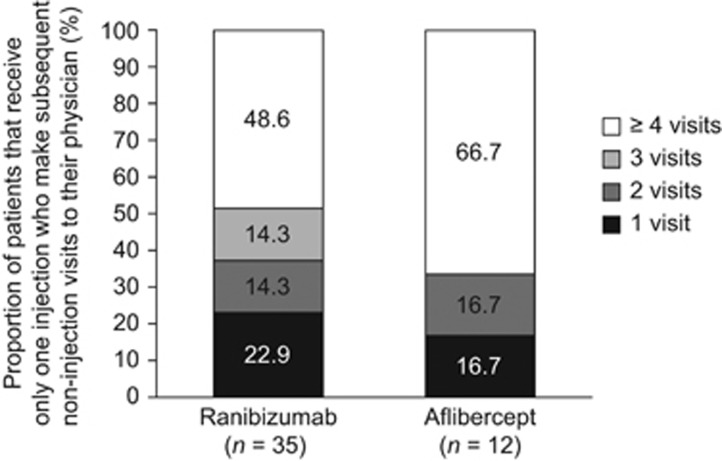
Number of visits by patients receiving only one injection.

Of patients receiving only one injection during follow-up (ranibizumab, *n*=35; aflibercept, *n*=12), 78.7% made more than one subsequent non-injection visit to their physician (ranibizumab, 77.1% aflibercept, 83.3% Figure 4). When comparing this subset of 47 patients who received only one injection during follow-up with those who received two or more injections, these results were not significantly associated with differences in sex, age, CCI, region, payer type, or the specialty of the prescribing physician, although CCI did approach significance (*P*=0.05).

The majority of the 285 patients included in the primary analysis received treatment in only one eye throughout follow-up, with only 3.5% of patients receiving bilateral treatment at any point during follow-up (ranibizumab, 3.9% aflibercept, 2.5%). When the inclusion criteria were relaxed to include any anti-VEGF treatment received during follow-up (*n*=354), 4.5% of patients were observed to have received bilateral treatment at some point during follow-up (ranibizumab, 4.6% aflibercept, 4.3%).

Of the 285 patients included in this study, none were found to have a claim relating to glaucoma associated with vascular disorders (ICD-9 365.63), diabetic macular edema (DME; ICD-9 362.07) or neovascular age-related macular degeneration (nAMD; ICD-9 362.50, 362.51, 362.52) in the 6 months before index. One patient had a recorded claim for DME within the first year after index; claims relating to glaucoma associated with vascular disorders and nAMD were not observed during follow-up.

## Discussion

This patient-level claims database analysis is, to our knowledge, the first to directly compare the patterns of ranibizumab and aflibercept use when given for treatment of CRVO. The main finding is that the number of injections received and total number of visits made by patients continuously treated with their index therapy was not significantly different regardless of whether patients started treatment with ranibizumab or aflibercept. There were no discernible demographic differences between patients in the ranibizumab and aflibercept groups.

In the USA, both the anti-VEGF treatments assessed presently are recommended to be given as monthly intravitreal injections for the management of macular edema secondary to CRVO. However, the presented results suggest that very few patients receive this regularity of injection throughout the first year of treatment. Potentially, this is due at least in part to improved visual outcomes in the patients receiving these anti-VEGF treatments, as has been seen in clinical trials. However, our time sensitivity analysis shows that even in the first 6 months of treatment most patients do not receive monthly anti-VEGF treatment as recommended by the labels of ranibizumab and aflibercept. Furthermore, we have shown that the likelihood of bilateral treatment is low; less than 5% of patients in both the groups were treated bilaterally in the year after index, compared with reports that ∼10% of those with unilateral CRVO will develop the condition bilaterally.^1^

The similarity of the injections given and total visits made by patients in the ranibizumab and aflibercept groups suggest that physicians may be using these treatments similarly in routine clinical practice. These results are in alignment with those observed in other ophthalmic indications, where the number of injections and total visits made were similar whether patients received ranibizumab or aflibercept. Another recent US claims database study in patients with nAMD reported that 5.8 (ranibizumab) and 5.5 (aflibercept) injections were given annually.^18^ Furthermore, when the results were extended to include the number of any intravitreal anti-VEGF injections received during follow-up, the mean number of injections received in the 12 months after index was similar between treatment groups and also similar to those received by patients receiving continuous treatment with their index drug, suggesting that the physicians may be using the two drugs interchangeably.

Despite previous reports that a significant proportion of patients with CRVO subsequently experience neovascular glaucoma,^19^ we found no reports of glaucoma associated with vascular disorders in the 6 months before index or during follow-up. Other comorbidities such as DME and nAMD were also rare during the study period, with only one observation of a DME code during the follow-up period. The absence of disease overlap indicates that the treatment patterns are representative of patients with unambiguously diagnosed CRVO.

Given that current market prices for ranibizumab and aflibercept are similar (US wholesale acquisition costs per 0.5 mg vial: ranibizumab, $1950; aflibercept, $1850)^20^ and that injections constitute the majority of the treatment costs associated with these treatments, the observation that the number of injections administered and physician visits for each treatment is very similar suggests that budgetary considerations for both treatments are likely to be similar in routine clinical practice. Therefore, the findings of this study represent important considerations for payers when evaluating the cost effectiveness of these treatments in the real world. These study findings also highlight that the way new therapies are used in practice may differ from recommendations based on the clinical trials, and emphasize the importance of this type of post approval observational study.

Owing to the relative recency of aflibercept approval and the number of inclusion/exclusion criteria required to product robust results, a large database with rapid upload of data was essential in order to generate sufficient data for analysis. The IDW is one of the largest claims-based databases in the USA, and 95% of claims are available for analysis within 3 weeks of submission. We believe this to be the largest observational study of its type to directly compare ranibizumab and aflibercept. Our sensitivity analyses support the main findings and suggest that the comparable observations made between these two treatment groups are not confounded by differences between groups in the first 6 months of treatment; of the 55–60% of patients receiving four or more injections in the first year after index treatment, over 40% in each group received these injections in the first 6 months of treatment. As no visual acuity data are available in the claims database, it is not possible to conclude that patients who only received one injection did not need additional ones. However, the fact that the vast majority of those patients had one or more follow-up visits post injection is reassuring.

There are several limitations in this study. Like any observational studies comparing two treatments, patient inclusion could be subject to selection bias, particularly if there are differences between patients receiving ranibizumab and those receiving aflibercept. However, the similarities of the available baseline characteristics between both the groups of patients suggest that this is not the case. Physicians' approaches to treating CRVO could differ from each other. One potential way to address the physician-level specific would be to have run a GEE model with multiple levels of cluster nesting (physician and patient). However, in this data set, 147 physicians injected both ranibizumab and aflibercept (average number of patients per physicians: 1.9); 118 (80.3%) of these physicians injected only one or two patients. Therefore, there was not sufficient data to run a meaningful analysis with two levels of nesting. As the aflibercept treatment pool grows, the analysis may become more meaningful. In addition, this study involves a relatively small sample size after application of the inclusion criteria, especially for aflibercept. However, the similarity of the injection and total visit results provide no suggestion that our findings are underpowered, and even with extended analyses the results are unlikely to demonstrate any clinically meaningful differences in injections or total visits. This study uses physician-entered claims codes to assess treatment patterns; as such, a risk of misclassification is inevitable, although we believe any such misclassification applies to both treatment groups equally. For example, the lack of neovascular glaucoma during the study period is unlikely to mean that no such events occurred, and more likely reflects that the diagnosis was unrecorded or missed. Last, we were unable to assess the effect of injection frequency on visual outcomes. This is beyond the scope of this type of study and requires further investigation.

In conclusion, this study is the first to directly compare treatment patterns of ranibizumab and aflibercept administered for the management of CRVO in routine clinical practice in the USA. The results suggest that these two therapies are not used as recommended by the labels in the USA and that patients receive similar numbers of injection regardless of the treatment with which they initiate therapy. Further studies are warranted to link these findings to visual outcomes and to evaluate whether the treatment patterns observed in this US study represent those in other countries.
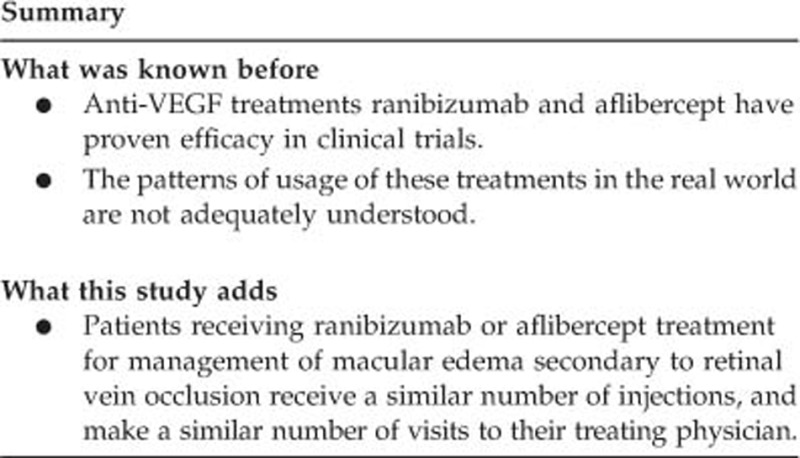


## Figures and Tables

**Table 1:** Baseline characteristics and patient demographics

	*Ranibizumab (*n=*206)*	*Aflibercept (*n=*79)*	P-value
Age, median years (interquartile range)	74.0 (67.0–81.0)	76.0 (70.0–81.0)	
*Sex, n (%)*			
Female	117 (57)	42 (53)	0.60^a^
Male	89 (43)	37 (47)	
			
*Prescriber of index medication,* n *(%)*			
Ophthalmologist	206 (100)	78 (99)	0.28^a^
Other	0 (0)	1 (1)	
*Health plan,* n *(%)*			
Medicaid	4 (2)	0 (0)	0.08^a^
Medicare	141 (68)	64 (81)	
Commercial	61 (30)	15 (19)	
			
*Geographic region,* n *(%)*			
Midwest	40 (19)	18 (23)	0.12^a^
Northeast	45 (22)	24 (30)	
South	107 (52)	29 (37)	
West	14 (7)	8 (10)	
			
*Charlson–Deyo comorbidities,* n *(%)*			
AIDS/HIV	0 (0)	0 (0)	
Cancer	17 (8)	4 (5)	
Chronic heart failure	6 (3)	4 (5)	
Chronic pulmonary disease	12 (6)	9 (11)	
Cardiovascular disease	10 (5)	5 (6)	
Dementia	1 (0)	1 (1)	
Diabetes with chronic complications	17 (8)	9 (11)	
Diabetes with or without acute complications	19 (9)	9 (11)	
Metastatic carcinoma	1 (0)	1 (1)	
Mild liver disease	0 (0)	1 (1)	
Moderate/severe liver disease	0 (0)	0 (0)	
Myocardial infarction	0 (0)	1 (1)	
Paraplegia/hemiplegia	0 (0)	0 (0)	
Peptic ulcer disease	0 (0)	0 (0)	
Peripheral vascular disease	1 (0)	3 (4)	
Renal disease	9 (4)	1 (1)	
Rheumatological disease	5 (2)	3 (4)	
Charlson–Deyo comorbidity index, mean (95% CI)	0.6 (0.5–0.8)	0.8 (0.5–1.1)	0.361^b^

Abbreviations: AIDS, acquired immunodeficiency syndrome; CI, confidence interval; HIV, human immunodeficiency virus.

a Statistical differences between categorical variables assessed using Fisher's exact test.

b Statistical differences between continuous variables assessed using unpaired Student's *t*-tests.

## Notes

Andrew Lotery has attended scientific advisory boards and received educational grants from Novartis Pharma AG, Basel, Switzerland and Bayer HealthCare AG, Leverkusen, Germany. Stephane Regnier is an employee of, and owns shares in, Novartis Pharma AG, Basel, Switzerland.

## References

[bib1] McIntosh RL Rogers SL Lim L Cheung N Wang JJ Mitchell P Natural history of central retinal vein occlusion: an evidence-based systematic review Ophthalmology 2010 117 (6 1113 1123 e1115 20430446 10.1016/j.ophtha.2010.01.060

[bib2] Rogers S McIntosh RL Cheung N Lim L Wang JJ Mitchell P The prevalence of retinal vein occlusion: pooled data from population studies from the United States, Europe, Asia, and Australia Ophthalmology 2010 117 (2 313 319 e311 20022117 10.1016/j.ophtha.2009.07.017 PMC2945292

[bib3] Evaluation of grid pattern photocoagulation for macular edema in central vein occlusion. The Central Vein Occlusion Study Group M report Ophthalmology 1995 102 (10 1425 1433 9097788 10.1016/s0161-6420(95)30849-4

[bib4] National Institute for Health and Care Excellence. TA 283. Ranibizumab for treating visual impairment caused by macular oedema secondary to retinal vein occlusion, 2013. Available at http://www.nice.org.uk/guidance/TA283 . Accessed on May 2014.

[bib5] Ip MS Scott IU VanVeldhuisen PC Oden NL Blodi BA Fisher M A randomized trial comparing the efficacy and safety of intravitreal triamcinolone with observation to treat vision loss associated with macular edema secondary to central retinal vein occlusion: the Standard Care vs Corticosteroid for Retinal Vein Occlusion (SCORE) study report 5 Arch Ophthalmol 2009 127 (9 1101 1114 19752419 10.1001/archophthalmol.2009.234 PMC2872173

[bib6] US Food and Drug Administration. Lucentis prescribing information, 2006. Available at http://www.gene.com/download/pdf/lucentis_prescribing.pdf . Accessed on July 2014.

[bib7] Kinge B Stordahl PB Forsaa V Fossen K Haugstad M Helgesen OH Efficacy of ranibizumab in patients with macular edema secondary to central retinal vein occlusion: results from the sham-controlled ROCC study Am J Ophthalmol 2010 150 (3 310 314 20591399 10.1016/j.ajo.2010.03.028

[bib8] Brown DM Campochiaro PA Singh RP Li Z Gray S Saroj N Ranibizumab for macular edema following central retinal vein occlusion: six-month primary end point results of a phase III study Ophthalmology 2010 117 (6 1124 1133 e1121 20381871 10.1016/j.ophtha.2010.02.022

[bib9] Thach AB Yau L Hoang C Tuomi L Time to clinically significant visual acuity gains after ranibizumab treatment for retinal vein occlusion: BRAVO and CRUISE trials Ophthalmology 2014 121 (5 1059 1066 24424249 10.1016/j.ophtha.2013.11.022

[bib10] Genentech. Press release: FDA approves Lucentis® (ranibizumab injection) for the treatment of macular edema following retinal vein occlusion, 2010. Available at http://www.gene.com/media/press-releases/12827/2010-06-22/fda-approves-lucentis-ranibizumab-inject . Accessed on June 2014.

[bib11] US Food and Drug Administration. Eylea prescribing information, 2011. Available at http://www.regeneron.com/Eylea/eylea-fpi.pdf . Accessed on July 2014.

[bib12] Korobelnik JF Holz FG Roider J Korobelnik JF Holz FG Roider J Intravitreal aflibercept injection for macular edema resulting from central retinal vein occlusion: one-year results of the phase 3 GALILEO study Ophthalmology 2014 121 (1 202 208 24084497 10.1016/j.ophtha.2013.08.012

[bib13] Holz FG Roider J Ogura Y Korobelnik JF Simader C Groetzbach G VEGF Trap-Eye for macular oedema secondary to central retinal vein occlusion: 6-month results of the phase III GALILEO study Br J Ophthalmol 2013 97 (3 278 284 23298885 10.1136/bjophthalmol-2012-301504

[bib14] Brown DM Heier JS Clark WL Boyer DS Vitti R Berliner AJ Intravitreal aflibercept injection for macular edema secondary to central retinal vein occlusion: 1-year results from the phase 3 COPERNICUS study Am J Ophthalmol 2013 155 (3 429 437 23218699 10.1016/j.ajo.2012.09.026

[bib15] Heier JS Clark WL Boyer DS Brown DM Vitti R Berliner AJ Intravitreal aflibercept injection for macular edema due to central retinal vein occlusion: two-year results from the COPERNICUS study Ophthalmology 2014 121 (7 1414 1420 24679444 10.1016/j.ophtha.2014.01.027

[bib16] Regeneron. Press release: Regeneron announces FDA approval of EYLEA® (aflibercept) injection for macular edema following central retinal vein occlusion, 2012. Available at http://investor.regeneron.com/releasedetail.cfm?ReleaseID=708835 . Accessed on July 2014.

[bib17] Deyo RA Cherkin DC Ciol MA Adapting a clinical comorbidity index for use with ICD-9-CM administrative databases J Clin Epidemiol 1992 45 (6 613 619 1607900 10.1016/0895-4356(92)90133-8

[bib18] Johnston SS Wilson K Huang A Smith D Varker H Turpcu A Retrospective analysis of first-line anti-vascular endothelial growth factor treatment patterns in wet age-related macular degeneration Adv Ther 2013 30 (12 1111 1127 24310208 10.1007/s12325-013-0078-4 PMC3906738

[bib19] Ryu CL Elfersy A Desai U Hessburg T Edwards P Gao H The effect of antivascular endothelial growth factor therapy on the development of neovascular glaucoma after central retinal vein occlusion: a retrospective analysis J Ophthalmol 2014 2014 317694 24800060 10.1155/2014/317694 PMC3995099

[bib20] Databank FAnalySource® Online. Available at http://www.fdbhealth.com/solutions/analysource-online-drug-pricing-software/ . Accessed on July 2014.

