# Supplementary material for: Patterns of ranibizumab and aflibercept treatment of central retinal vein occlusion in routine clinical practice in the USA
Source: Eye (Lond). 2015 Jan 9;29(3):380–7. doi: 10.1038/eye.2014.308 (PMC4366471; doi:10.1038/eye.2014.308)
Supplement: Supplementary Tables [file eye2014308x1.doc]

## Supplementary Table 1. Negative binomial model of the number of injections, non-injection visits and total visits by patients on anti-VEGF treatment for CRVO (*n =*285).

|  | **Injection visits** | | **Total visits** | | **Monitoring visits** | |
| --- | --- | --- | --- | --- | --- | --- |
|  | **Coefficient** | ***P* value** | **Coefficient** | ***P* value** | **Coefficient** | ***P* value** |
| **Drug** |  |  |  |  |  |  |
| Aflibercept | 0.074 | 0.376 | -0.043 | 0.515 | -0.245 | 0.058 |
| Ranibizumab (reference category) |  |  |  |  |  |  |
| **Age, years** |  |  |  |  |  |  |
| < 65 | 0.006 | 0.972 | -0.006 | 0.962 | -0.009 | 0.974 |
| 65–69 | -0.210 | 0.169 | -0.169 | 0.157 | -0.088 | 0.706 |
| 70–4 | -0.054 | 0.681 | -0.006 | 0.956 | 0.062 | 0.759 |
| 75–79 | -0.024 | 0.856 | 0.04 | 0.692 | 0.144 | 0.472 |
| 80–84 | -0.129 | 0.358 | -0.012 | 0.911 | 0.180 | 0.390 |
| ≥ 85 (reference category) |  |  |  |  |  |  |
| **Sex** |  |  |  |  |  |  |
| Female | -0.071 | 0.358 | -0.059 | 0.321 | -0.038 | 0.745 |
| Male (reference category) |  |  |  |  |  |  |
| **Charlson–Deyo Comorbidity Index** | 0.000 | 0.989 | 0.002 | 0.925 | 0.006 | 0.904 |
| **Health plan type** |  |  |  |  |  |  |
| Medicaid | -0.283 | 0.415 | -0.119 | 0.64 | 0.113 | 0.807 |
| Medicare | -0.003 | 0.976 | -0.03 | 0.721 | -0.064 | 0.694 |
| Commercial (reference category) |  |  |  |  |  |  |
| **Geographic region** |  |  |  |  |  |  |
| Midwest | -0.051 | 0.612 | 0.08 | 0.288 | 0.281 | 0.048 |
| Northeast | 0.034 | 0.715 | 0.048 | 0.509 | 0.069 | 0.624 |
| West | -0.042 | 0.773 | -0.09 | 0.435 | -0.142 | 0.532 |
| South (reference category) |  |  |  |  |  |  |

CRVO, central retinal vein occlusion. VEGF, vascular endothelial growth factor.

## Supplementary Table 2. Generalized estimating equation model of interval between doses of anti-VEGF for CRVO (all patients who received two or more injections [*n =*238]).

|  | **Coefficient** | ***P* value** |
| --- | --- | --- |
| **Drug** |  |  |
| Aflibercept | -0.042 | 0.443 |
| Ranibizumab (reference category) |  |  |
| **Age, years** |  |  |
| < 65 | 0.154 | 0.119 |
| 65–69 | 0.194 | 0.030 |
| 70–74 | 0.195 | 0.009 |
| 75–79 | 0.165 | 0.015 |
| 80–84 | 0.166 | 0.023 |
| ≥ 85 (reference category) |  |  |
| **Sex** |  |  |
| Female | 0.051 | 0.363 |
| Male (reference category) |  |  |
| **Charlson–Deyo Comorbidity Index** | -0.011 | 0.577 |
| **Health plan type** |  |  |
| Medicaid | -0.099 | 0.691 |
| Medicare | 0.035 | 0.672 |
| Commercial (reference category) |  |  |
| **Geographic region** |  |  |
| Midwest | 0.022 | 0.752 |
| Northeast | 0.047 | 0.428 |
| West | 0.056 | 0.709 |
| South (reference category) |  |  |

CRVO, central retinal vein occlusion. VEGF, vascular endothelial growth factor.
